# A Comparative Analysis of Comorbidities, Clinical Profiles, and Treatment Outcomes Among Geriatric and Non-geriatric Tuberculosis Patients in New Delhi, India: A Retrospective Cohort Study

**DOI:** 10.7759/cureus.105771

**Published:** 2026-03-24

**Authors:** Shweta Gupta, Vyomesh Rastogi, Divyendu Sharma, Mitasha Singh, Vaibhav Sharawat, Raj Nandini, Ravi P Jha, Kevika Goel, Shubhankar Das

**Affiliations:** 1 Pulmonary and Critical Care Medicine, Dr. Baba Saheb Ambedkar Medical College and Hospital, New Delhi, IND; 2 Community Medicine, Dr. Baba Saheb Ambedkar Medical College and Hospital, New Delhi, IND; 3 Biostatistics and Epidemiology, Dr. Baba Saheb Ambedkar Medical College and Hospital, New Delhi, IND; 4 Biological Sciences, Sri Venkateswara College, University of Delhi, New Delhi, IND

**Keywords:** comorbidity, determinant, geriatrics, india, national tuberculosis elimination program (ntep), retrospective cohort study, risk factors, tb, treatment outcome, tuberculosis

## Abstract

Background

Tuberculosis (TB) outcomes are strongly influenced by age-related factors and coexisting chronic illnesses. With a rapidly aging population and a rising non-communicable disease burden in India, understanding how comorbidities affect clinical presentation and treatment outcomes among geriatric TB patients is essential for optimizing programmatic care under the National Tuberculosis Elimination Programme (NTEP).

Methods

This retrospective comparative cohort study was conducted at a tertiary care hospital in New Delhi and included 951 adult TB patients enrolled under NTEP between March and August 2025. A retrospective cohort design was adopted, as exposure (age group and comorbidity status) and outcomes (treatment outcomes) had already occurred and were assessed using existing programmatic records. Patients were categorized into geriatric (≥60 years) and non-geriatric (18-60 years) groups. Demographic characteristics, comorbidities, clinical profiles, diagnostic modalities, and treatment outcomes were extracted from DOTS (Directly Observed Treatment, Short-course) records and the NIKSHAY platform. Associations between age group and categorical variables were assessed using the Chi-square and Fisher’s exact tests, while continuous variables were compared using independent-sample t-tests. A multinomial logistic regression model, with death as the reference category, was used to identify independent predictors of treatment outcome across three groups: treatment success, treatment failure/loss to follow-up, and death.

Results

Of the 951 patients analyzed, 433 were geriatric and 518 were non-geriatric. Geriatric patients were more frequently male (68.8% vs. 56.9%; p < 0.001) and had a significantly higher burden of comorbidities (67.4% vs. 22.8%; p < 0.001), particularly diabetes mellitus (DM) (35.1% vs. 16.2%; p < 0.001). Pulmonary TB predominated in the geriatric group (84.3%), while extrapulmonary TB was more common among non-geriatric patients (43.8%; p < 0.001). Microbiological confirmation was higher in geriatric patients (72.5% vs. 46.1%; p < 0.001). Treatment success was significantly lower among geriatric patients (62.0% vs. 87.8%), while mortality (17.3% vs. 3.3%) and loss to follow-up/treatment failure (20.8% vs. 8.9%) were markedly higher (p < 0.001). On multinomial logistic regression, geriatric age was the strongest independent predictor of treatment failure, with geriatric patients having approximately 85% lower odds of achieving treatment success, compared to death, relative to non-geriatric patients (aOR = 0.148; 95% CI: 0.076-0.289; p < 0.001). Non-reactive human immunodeficiency virus (HIV) status was independently associated with significantly higher odds of treatment success (aOR = 4.644; 95% CI: 2.459-8.772; p < 0.001).

Conclusion

Geriatric TB patients exhibit a significantly higher comorbidity burden, greater pulmonary involvement, and markedly poorer treatment outcomes than younger adults. Geriatric age is an independent predictor of mortality, underscoring the urgent need for age-specific treatment strategies, intensified monitoring, and targeted comorbidity management within NTEP to improve outcomes in this vulnerable population.

## Introduction

Tuberculosis (TB) remains one of the most significant global public health challenges, despite advances in diagnostics, treatment, and control programs. According to the World Health Organization (WHO) Global Tuberculosis Report 2025, an estimated 10.7 million people developed TB in 2024, with approximately 1.23 million deaths worldwide [1]. Although improvements in case detection and treatment access have occurred following the COVID-19 pandemic, global progress remains insufficient to achieve the targets set under the WHO End Tuberculosis Strategy. The disease burden continues to be concentrated in a limited number of high-burden countries, with nearly 87% of cases occurring in 30 nations, and India alone accounting for approximately one-quarter of the global TB incidence [1].

In response to this substantial burden, India’s National Tuberculosis Elimination Programme (NTEP) has implemented major programmatic reforms, including expanded molecular diagnostic capacity, enhanced screening strategies, digital surveillance systems, and improved treatment coverage. As a result, India reported 2.61 million TB notifications in 2024 - the highest number recorded to date - reflecting improved surveillance and case detection [2]. However, TB outcomes remain strongly influenced by underlying comorbid conditions and social determinants of health. Key risk factors, such as undernutrition, diabetes mellitus (DM), human immunodeficiency virus (HIV) infection, tobacco use, and alcohol use disorders, substantially contribute to the global TB burden [3]. Among these, DM is particularly important, increasing the risk of active TB by two- to four-fold and contributing to delayed sputum conversion, higher relapse rates, and increased mortality [4,5].

The intersection of TB with chronic diseases is becoming increasingly important in India, due to demographic transition and rising life expectancy. Older adults represent a particularly vulnerable population because of immunosenescence, malnutrition, frailty, polypharmacy, and the presence of multiple comorbidities. Studies from India indicate that individuals aged ≥60 years may account for 10%-20% of TB cases in tertiary-care settings and frequently present with atypical or less specific symptoms, which can delay diagnosis [6-8]. In addition, chronic conditions, such as DM, chronic obstructive pulmonary disease (COPD), cardiovascular disease, chronic kidney disease, anemia, and undernutrition, are more prevalent among elderly individuals and may significantly influence disease presentation and treatment response.

Treatment outcomes among elderly TB patients are often less favorable than those observed in younger adults. Increased mortality, higher rates of adverse drug reactions, delayed sputum conversion, and greater need for hospitalization have been reported in older populations, despite comparable treatment adherence [9]. These clinical challenges are often compounded by socioeconomic vulnerabilities, including limited financial resources, reduced health-seeking behavior, and dependency for daily activities.

Despite growing recognition of these issues, contemporary Indian evidence comparing the burden and impact of comorbidities between geriatric and non-geriatric TB patients remains limited. Many existing studies are cross-sectional, based on older datasets, or do not adequately stratify outcomes by age group, or evaluate the cumulative effects of multimorbidity and nutritional status. Furthermore, while NTEP programmatic data increasingly highlight the coexistence of chronic diseases among TB patients, age-specific approaches for risk stratification and clinical management remain insufficiently defined.

Therefore, the present study aims to conduct a comparative evaluation of geriatric (≥60 years) and non-geriatric (<60 years) TB patients enrolled under the NTEP at a tertiary care hospital in New Delhi. The primary objective is to determine whether geriatric age is an independent determinant of treatment outcomes under NTEP, particularly treatment success, mortality, and overall unfavorable outcomes, and to identify independent predictors of adverse treatment outcomes using multivariable analysis, with emphasis on age and coexisting chronic illnesses. The secondary objectives are to compare the comorbidity burden and multimorbidity patterns, as well as clinical presentation and diagnostic characteristics, between geriatric and non-geriatric TB patients, and to examine the relationship between multimorbidity and disease presentation or treatment response. By providing age-stratified evidence on the clinical and prognostic impact of comorbidities in TB, the study seeks to inform risk-stratification strategies and geriatric-sensitive approaches within programmatic TB care, thereby supporting India’s TB elimination efforts.

## Materials and methods

This retrospective-comparative cohort study was conducted to evaluate differences in clinical presentation, comorbidities, and treatment outcomes between geriatric patients (aged >60 years) and non-geriatric adults (18-60 years) diagnosed with pulmonary TB. The study was carried out at the Directly Observed Treatment, Short-Course (DOTS) Centre, at the Department of Respiratory Medicine of a tertiary-care teaching hospital in New Delhi, India. The study population consisted of all patients registered at the DOTS Centre during the period from March 1, 2025, to August 31, 2025. Eligible participants were categorized into two cohorts based on age: Group 1 (geriatric cohort) included patients older than 60 years diagnosed with TB, while Group 2 (non-geriatric cohort) included patients aged between 18 and 60 years with TB. Patients younger than 18 years and those diagnosed with multidrug-resistant tuberculosis (MDR-TB) were excluded from the study.

**Figure 1:**
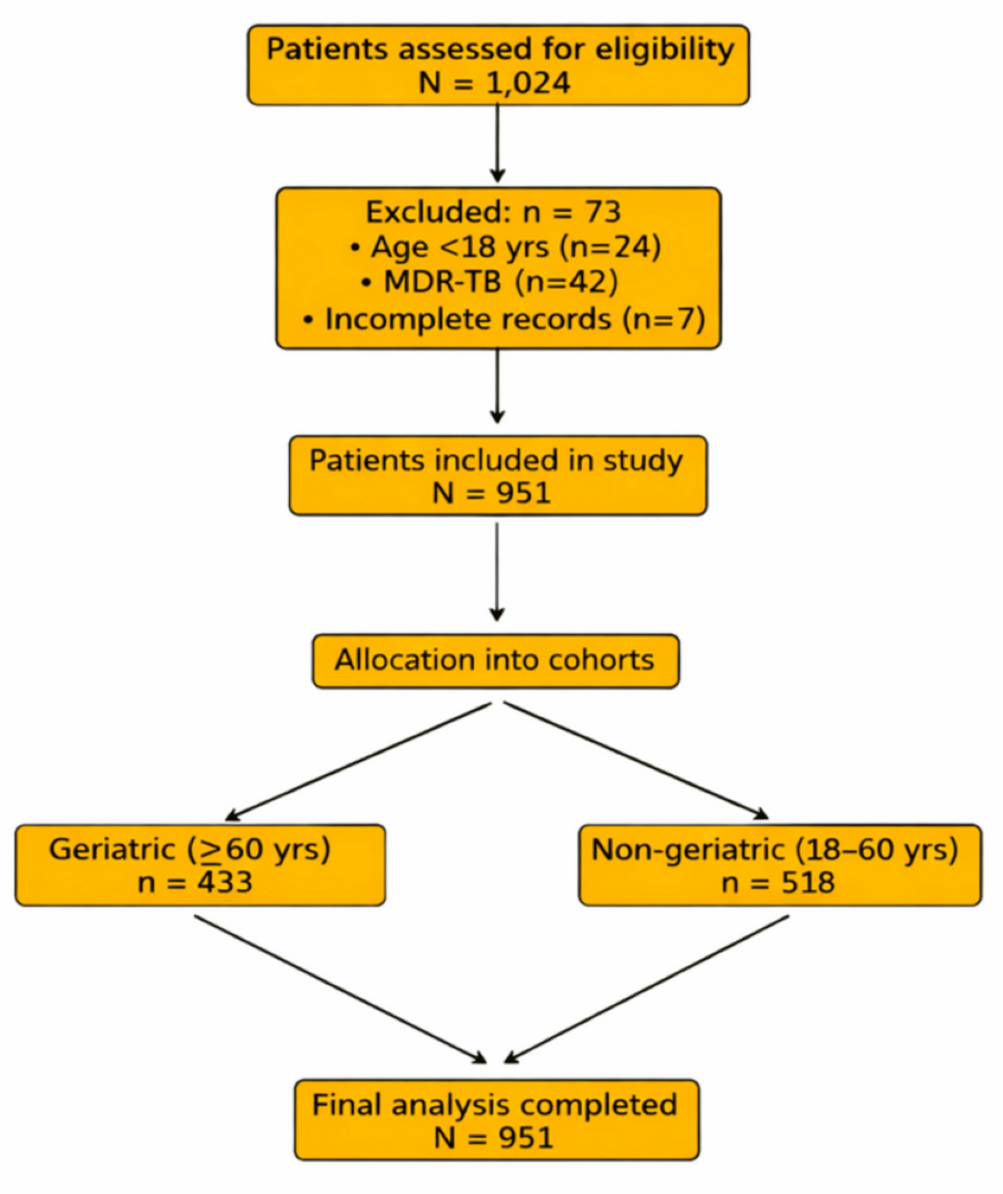
Study Flowchart

The sample size was calculated using anticipated rates of unfavorable outcomes for comparing two proportions, reported in previous literature, using the formula stated as: $N_{1}=\left\{z_{1-\alpha / 2} * \sqrt{\bar{p} * \bar{q} *\left(1+\frac{1}{k}\right)}+z_{1-\beta} * \sqrt{p_{1} * q_{1}+\left(\frac{p_{2} * q_{2}}{k}\right)}\right\}^{2} / \Delta^{2}$, where p1 and p2 represent the expected proportions of unfavorable treatment outcomes in geriatric and non-geriatric groups, respectively, and q = 1 - p. p̄ denotes the average proportion across both groups. z_1-α/2_ corresponds to the 95% confidence level, z_1-β_ represents the desired statistical power, k is the ratio between the two groups, and Δ is the minimum detectable difference in outcome proportions. These parameters were selected based on previously reported proportions of unfavorable TB treatment outcomes. Based on findings by Murali et al. [10], the expected incidence of unfavorable treatment outcomes was 12% in geriatric and 6.5% in non-geriatric TB populations. Using a Type I error of 5% and a Type II error of 20% (power 80%), the minimum calculated sample size was 866 participants (433 per group). However, a larger number of patients were included in the non-geriatric cohort, resulting in a total sample size of 951 patients (433 geriatric and 518 non-geriatric), consistent with Figure 1. 

Data were collected retrospectively from the DOTS treatment cards and the NIKSHAY digital platform [11]. Each DOTS card included demographic information, socioeconomic characteristics, disease category, sputum smear microscopy results, diagnostic tests, HIV serostatus, diabetes status, and treatment regimen details. Chest radiograph findings were extracted from routine clinical records, where radiographs were interpreted and documented by the treating clinicians as part of standard TB program practice, rather than through standardized radiologist reporting. The NIKSHAY system was accessed using each patient’s unique NIKSHAY identification number to retrieve additional information on treatment adherence, follow-up sputum results, and final treatment outcomes. Additionally, patients were individually contacted telephonically to verify comorbidity status and confirm treatment outcomes when such information was not explicitly documented in the DOTS treatment cards or the NIKSHAY platform. 

Comorbidities were identified from NTEP/NIKSHAY records and corresponding patient case files. DM was defined as a documented diagnosis in medical records, ongoing anti-diabetic treatment, or recorded fasting plasma glucose ≥126 mg/dL, or random blood glucose ≥200 mg/dL at baseline evaluation. Hypertension was defined as a documented diagnosis, use of antihypertensive medications, or recorded blood pressure ≥140/90 mmHg during at least one clinical visit, as per available records. HIV infection status was based on confirmed positivity documented in NIKSHAY records, following the Integrated Counselling and Testing Centre (ICTC) or other diagnostic testing. Chronic kidney disease was defined by a prior documented diagnosis or evidence of persistently deranged renal function noted by the treating physician. Chronic respiratory diseases, including COPD, asthma, or bronchiectasis, were identified based on prior documented diagnoses or supportive clinical and radiological evidence in case files. Other comorbidities were defined according to physician-documented diagnoses available within programmatic records.

Outcome definitions

Treatment outcomes were defined according to the standardized programmatic definitions of the NTEP. Treatment success was defined as the sum of patients categorized as cured or treatment completed at the end of therapy. Treatment failure referred to patients whose sputum smear or culture remained positive at five months or later during treatment, or to those requiring a change in treatment regimen due to lack of response. Mortality (death) was defined as death from any cause during the course of anti-tubercular therapy. Loss to follow-up (LFU) referred to patients whose treatment was interrupted for one month or more after initiation of therapy. These standardized definitions were used to ensure consistency in outcome classification across the study population.

All data were coded and entered in a Microsoft Excel spreadsheet (Microsoft® Corp., Redmond, WA, USA) before being imported into IBM SPSS Statistics for Windows, Version 29 (Released 2022; IBM Corp., Armonk, NY, USA) for statistical analysis. Qualitative variables, such as comorbidities and treatment outcomes, were presented as frequencies and percentages. Quantitative data, such as age and weight, were summarized using means and standard deviations. Associations between age group (geriatric vs. non-geriatric) and categorical variables were assessed using the Chi-square test and Fisher's exact test, while continuous variables were compared using independent sample t-tests. To identify independent predictors of treatment outcome, a multinomial logistic regression analysis was performed, with treatment outcome as the dependent variable comprising three categories: treatment success, treatment failure/LFU, and death, with death serving as the reference category. Socio-demographic and clinical parameters with clinically relevant covariates, including age group, sex, weight, HIV status, diabetes status, socioeconomic status, site of disease, programmatic classification of TB case, and presence of comorbidities, were entered into the regression model. Results were expressed as adjusted odds ratios (aORs) with their corresponding 95% confidence intervals (CI), and a p-value of less than 0.05 was considered statistically significant.

To ensure data validity, quality assurance measures were undertaken throughout the study period. Study investigators reviewed patient eligibility, verified the completeness of extracted information, cross-checked NIKSHAY data with DOTS records, and ensured prevention of duplicate entries. Data entry underwent periodic verification by a second reviewer to minimize potential errors. The study followed strict confidentiality measures, as per the Indian Council of Medical Research (ICMR) guidelines. Ethical approval for this study was obtained from both the Institutional Research Review Committee (IRRC) and the Institutional Ethics Committee (IEC) of the institution, as of August 31, 2022, with Ethical Approval Number 5(32)/2020/BSAH/DNB/PF/22608/09. The study adhered to principles outlined in the ICMR National Ethical Guidelines for Biomedical and Health Research involving Human Participants. As the study relied solely on existing patient records and involved no direct interaction or intervention, the requirement for informed consent was waived by the ethics committee.

## Results

Sociodemographic and clinical characteristics

A total of 951 TB patients were included in the study, of whom 518 (54.5%) were non-geriatric (mean age 31.35 ± 11.14) and 433 (45.5%) were geriatric (aged ≥60 years; mean age 66.72 ± 6.38). Among geriatric patients, males predominated significantly (68.8% vs. 56.9% in non-geriatric patients; p < 0.001). The prevalence of comorbidities was markedly higher in the geriatric group (67.4%) compared to the non-geriatric group (22.8%; p < 0.001). Diabetes was present in 35.1% of geriatric patients versus 16.2% of non-geriatric patients (p < 0.001). HIV-reactive status was more frequent among non-geriatric patients (4.6% vs. 1.2%; p < 0.001), while a greater proportion of geriatric patients had unknown HIV status (21.7% vs. 13.5%). Pulmonary TB predominated in geriatric patients (84.3%), whereas extrapulmonary TB was more common among non-geriatric patients (43.8%; p < 0.001). Microbiological confirmation was achieved in 72.5% of geriatric patients, compared to 46.1% of non-geriatric patients (p < 0.001). Sputum culture was the predominant diagnostic modality in geriatric patients (21.7%), while sputum microscopy and clinical diagnosis were more evenly distributed across both groups. Treatment success was significantly lower among geriatric patients (62.0%) compared to non-geriatric patients (87.8%). Mortality was substantially higher in the geriatric group (17.3% vs. 3.3%), and LFU/treatment failure was also more frequent among geriatric patients (20.8% vs. 8.9%; p < 0.001 for all).

Marked differences were observed in the diagnostic profiles of both cohorts. The clinical and demographic comparison between geriatric and non-geriatric TB patients is shown in Table 1.

**Table 1:** Clinical and Demographic Comparison Between Geriatric and Non-geriatric Tuberculosis Patients n: number; χ²: Chi-square test; p: p-value. *Chi-square test; ^independent-sample t test; #Chi-square test with continuity correction. Treatment success = cured + treatment completed. CBNAAT: Cartridge-Based Nucleic Acid Amplification Test; LPA: Line Probe Assay; TB: Tuberculosis; PTB: Pulmonary TB; EPTB: Extra-Pulmonary TB; HIV: Human Immunodeficiency Virus; LTFU: Loss to Follow-Up; TF/LFU: Treatment Failure/Loss to Follow-Up; NA: Not Applicable

Variable	Non-geriatric (n = 518)	Geriatric (n = 433)	Test Statistic, p-value
Gender			
Female	223 (43.1%)	135 (31.2%)	14.16, <0.001*
Male	295 (56.9%)	298 (68.8%)	
Weight (mean ± SD)	48.86 (10.96)	50.10 (12.29)	1.62, 0.10^
Socioeconomic status			
Lower	12 (2.3)	8 (1.8)	2.25, 0.52*
Lower middle	207 (40.0)	176 (40.6)	
Upper lower	225 (43.4)	174 (40.2)	
Upper middle	74 (14.3)	75 (17.3)	
Comorbidities			
Yes	118 (22.8%)	292 (67.4%)	191.77, <0.001*
No	400 (77.2%)	141 (32.6%)	
HIV status			
Non-reactive	424 (81.9%)	334 (77.1%)	19.20, <0.001*
Reactive	24 (4.6%)	5 (1.2%)	
Unknown	70 (13.5%)	94 (21.7%)	
Diabetes status			
Diabetic	84 (16.2%)	152 (35.1%)	73.91, <0.001*
Non-diabetic	421 (81.3%)	242 (55.9%)	
Unknown	13 (2.5%)	39 (9.0%)	
Basis of diagnosis			
CBNAAT	72 (13.9%)	58 (13.4%)	142.92, <0.001*
Clinical TB	184 (35.5%)	150 (34.6%)	
Molecular	96 (18.5%)	3 (0.7%)	
Sputum culture	16 (3.1%)	94 (21.7%)	
Sputum microscopy	150 (29.0%)	128 (29.6%)	
Microbiologically confirmed			
No	279 (53.9%)	119 (27.5%)	67.44, <0.001*
Yes	239 (46.1%)	314 (72.5%)	
Type of case			
New	445 (85.9%)	292 (67.4%)	105.95, <0.001*
Recurrent	42 (8.1%)	38 (8.8%)	
Treatment after failure	8 (1.5%)	9 (2.1%)	
Treatment after LFU	23 (4.4%)	15 (3.5%)	
Previously treated	0	79 (18.2%)	
Site of disease			
Extra pulmonary	227 (43.8%)	68 (15.7%)	87.14, <0.001*
Pulmonary	291 (56.2%)	365 (84.3%)	
Treatment interruptions			
Yes	21 (4.1%)	0 (0.0%)	16.12, <0.001#
No	497 (95.9%)	433 (100.0%)	
Final treatment outcome			
Treatment success	455 (87.8%)	230 (62.0%)	87.06, <0.001*
Died	17 (3.3%)	64 (17.3%)	
Treatment failure/loss to follow-up	46 (8.9%)	77 (20.8%)	

The burden of comorbidities was markedly greater among geriatric patients, with 67.4% having at least one comorbidity, compared to 22.8% in the non-geriatric group (χ² = 191.773, p < 0.001). DM was the most prevalent condition and was significantly more common in the elderly (35.3% vs. 16.2%; χ² = 73.914, p < 0.001). Other comorbidities were also more frequent in the geriatric cohort, including hypertension (18.5% vs. 4.2%), chronic kidney disease (5.5% vs. 2.5%), asthma (7.2% vs. 0.8%), and chronic obstructive pulmonary disease (3.8% vs. 1.7%). Multiple comorbidities were seen in 18.8% of geriatric patients, compared to 0.8% of non-geriatric patients. In contrast, among the 518 non-geriatric TB patients, 118 (22.8%) had at least one documented comorbidity. Within this subgroup, chronic liver disease (CLD) was the most frequently recorded comorbidity, observed in 60 patients (50.8% of those with comorbidities), corresponding to 11.6% of the total non-geriatric cohort. In comparison, CLD was present in 2.7% of geriatric patients. Additionally, HIV positivity was significantly higher among non-geriatric patients (4.6% vs. 1.2%; χ² = 19.203, p < 0.001), highlighting a distinct comorbidity pattern between the two age groups. The distribution of comorbid conditions across both study groups is detailed in Table 2.

**Table 2:** Distribution of Comorbidities Among Geriatric and Non-geriatric Tuberculosis Patients Note: “Multiple” indicates the presence of more than one comorbid condition in a single patient. “No comorbidities” refers to patients without any documented coexisting medical condition. Percentages are calculated within each age group. COPD: Chronic Obstructive Pulmonary Disease; TB: Tuberculosis

Age Group	Comorbidity	Frequency	Percent
Non-geriatric	Asthma	1	0.2
	Chronic kidney disease	3	0.6
	Chronic liver disease	60	11.6
	COPD	2	0.4
	Diabetes mellitus	36	6.9
	Hypertension	5	1
	Hypothyroidism	10	1.9
	Multiple	1	0.2
	Total (with comorbidities)	118	22.8
	No comorbidities	400	77.2
	Total	518	100
Geriatric	Asthma	21	4.8
	Chronic kidney disease	16	3.7
	Chronic liver disease	8	1.8
	COPD	11	2.5
	Diabetes mellitus	103	23.8
	Hypertension	54	12.5
	Hypothyroidism	8	1.8
	Ischemic heart disease	16	3.7
	Multiple	55	12.7
	Total (with comorbidities)	292	67.4
	No comorbidities	141	32.6
	Total	433	100

**Figure 2:**
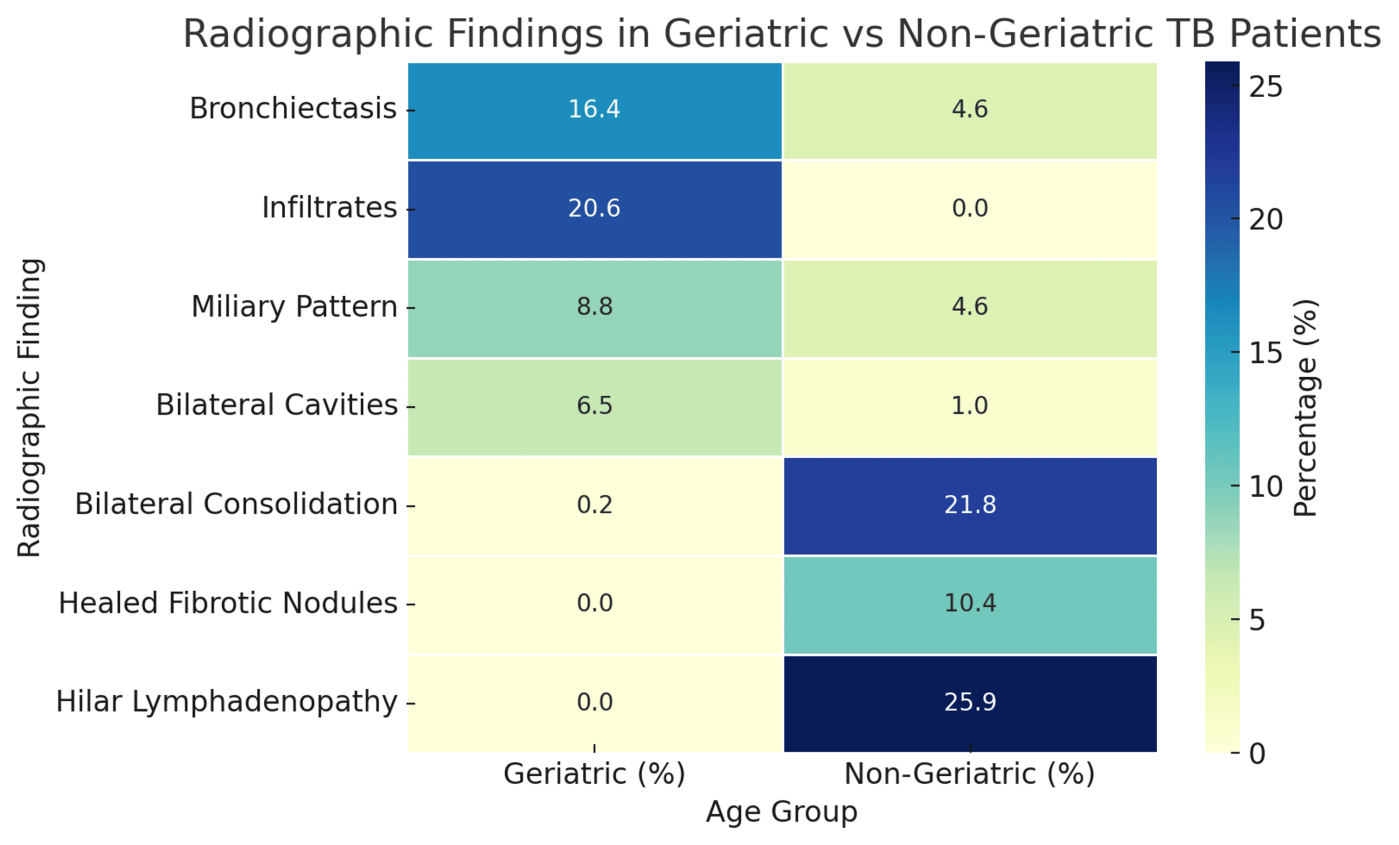
Radiographic Findings in Geriatric and Non-geriatric Tuberculosis Patients Heatmap illustrating the radiographic findings in geriatric vs. non-geriatric tuberculosis (TB) patients - bronchiectasis, infiltrates, miliary pattern, and bilateral cavities are more frequent in the elderly, whereas non-geriatric patients show higher rates of consolidation, healed nodules, and hilar lymphadenopathy. Radiographic findings revealed a higher prevalence of bronchiectasis and infiltrates among the elderly (χ² = 465.039, p < 0.001).

**Figure 3:**
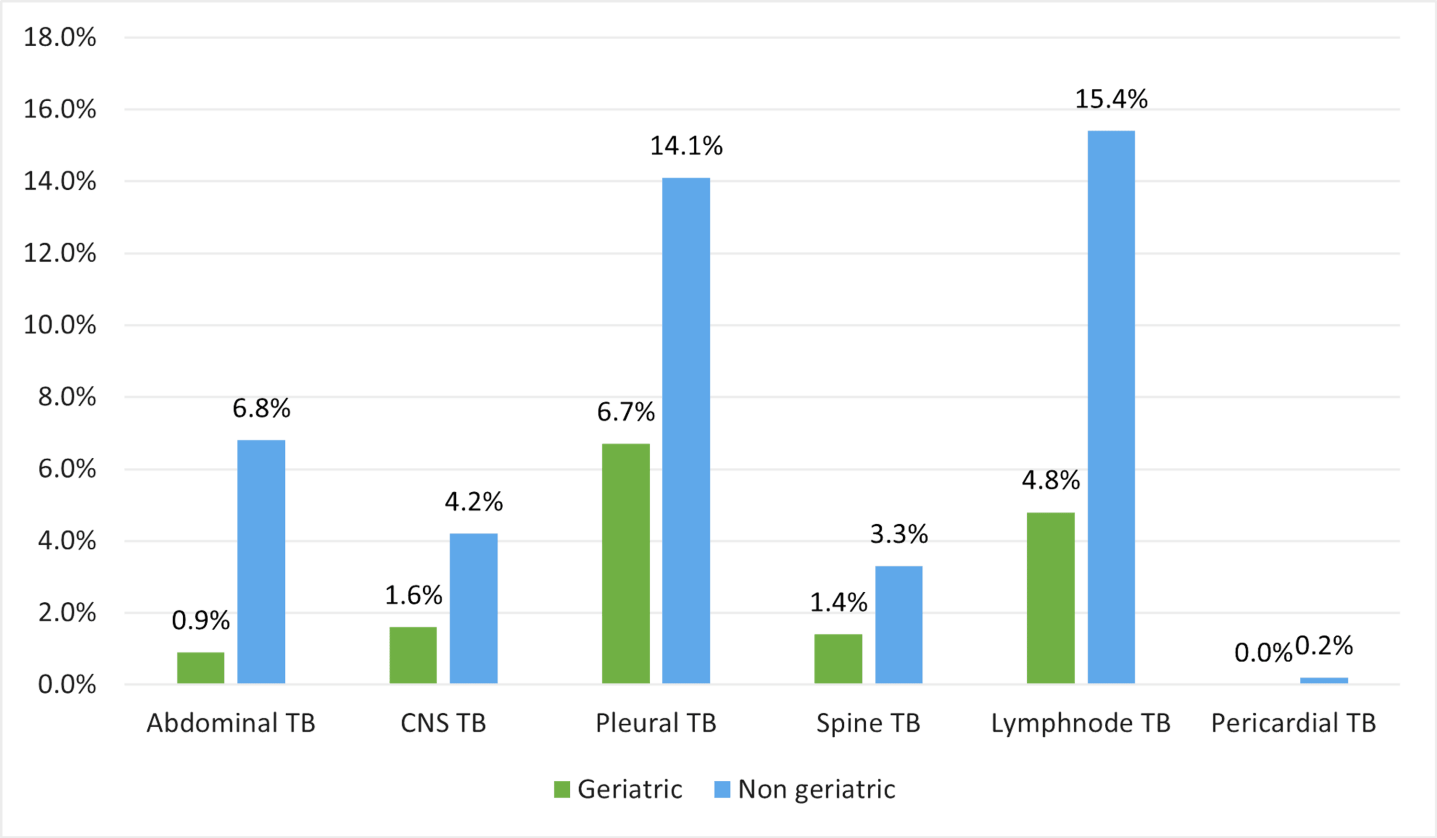
Comparison of Site-Wise Distribution of Extrapulmonary TB Between Geriatric and Non-geriatric Patients Note: The bar chart illustrates the percentage distribution of different forms of extrapulmonary TB among geriatric and non-geriatric patients. Categories include abdominal, CNS, pleural, spinal, lymph node, and pericardial TB. Percentages are calculated within each age group. TB: Tuberculosis; CNS TB: Central Nervous System Tuberculosis

Radiographic findings differed considerably between the groups. Elderly patients more often presented with bronchiectasis (16.4% vs. 4.6%), infiltrates (20.6% vs. 0%), miliary patterns (8.8% vs. 4.6%), and bilateral cavities (6.5% vs. 1.0%). Non-geriatric patients were more likely to exhibit bilateral consolidations (21.8% vs. 0.2%), healed fibrotic nodules (10.4% vs. 0%), and hilar lymphadenopathy (25.9% vs. 0%). All chest X-ray findings are showcased as a heatmap in Figure 2. Among extrapulmonary sites, tubercular lymphadenopathy was the most frequent in both age groups, followed by pleural effusion and abdominal TB (Figure 3).

Multinomial logistic regression analysis

A multinomial logistic regression was performed with death as the reference outcome category and three outcome groups: treatment success, treatment failure/LFU, and death (Table 3).

**Table 3:** Multinomial Logistic Regression Analysis of Factors Associated With Treatment Outcomes (Treatment Success and Treatment Failure/Loss to Follow-Up Compared to Death) Among Tuberculosis Patients Note: "Ref" category is for dead patients. Adj OR: Adjusted Odds Ratio; CI: Confidence Interval; HIV: Human Immunodeficiency Virus; TB: Tuberculosis; LFU: Loss to Follow-Up; EPTB: Extrapulmonary Tuberculosis; PTB: Pulmonary Tuberculosis; Ref: Reference Category; kg: Kilograms

Outcome-Treatment Success	p-value	Adj OR	95% Confidence Interval Adj OR	
			Lower Bound	Upper Bound
Weight in kg	0.21	1.01	0.99	1.04
Sex				
Female	0.61	0.87	0.51	1.48
Male	Ref	-	-	-
Socioeconomic status score				
Lower	0.09	0.28	0.06	1.25
Lower middle	0.51	0.76	0.34	1.69
Upper lower	0.97	0.98	0.44	2.20
Upper middle	Ref	-	-	-
Age-wise distribution				
Geriatric	<0.001	0.15	0.07	0.29
Non-geriatric	Ref	-	-	-
HIV status				
Non-reactive	<0.001	4.644	2.459	8.77
Reactive	0.16	4.602	0.546	38.78
Unknown	Ref	-	-	-
Diabetes				
Present	0.75	1.19	0.39	3.61
Non-diabetic	0.67	1.27	0.42	3.82
Unknown	Ref	-	-	-
Programmatic classification of TB case				
New case	0.88	1.10	0.31	3.97
Recurrent	0.92	0.92	0.20	4.21
Treatment after failure	0.28	2.44	0.48	12.46
Treatment after loss to follow-up	0.15	0.27	0.05	1.58
Previously treated	Ref	-	-	-
Comorbidity				
None	0.28	1.36	0.77	2.37
Present	Ref	-	-	-
Site-wise classification				
EPTB	0.65	1.16	0.61	2.20
PTB	Ref	-	-	-
Outcome-Treatment Failure/Loss to Follow-Up	p-value	Adj OR	95% CI for OR - Lower Bound	Upper Bound
Weight in kg	0.21	1.02	0.99	1.04
Socioeconomic status score				
Lower	0.44	0.53	0.10	2.71
Lower middle	0.68	0.83	0.34	2.03
Upper lower	0.59	0.77	0.31	1.94
Upper middle	Ref	-	-	-
Sex				
Female	0.81	0.93	0.51	1.71
Male	Ref	-	-	-
Age-wise distribution				
Geriatric	0.12	0.54	0.25	1.16
Non-geriatric	Ref	-	-	-
HIV status				
Non reactive	0.46	1.30	0.64	2.64
Reactive	0.40	2.68	0.26	27.33
Unknown	Ref	-	-	-
Diabetes				
Present	0.84	0.88	0.26	2.97
Non-diabetic	0.76	1.21	0.36	4.08
Unknown	Ref	-	-	-
Programmatic classification of TB case				
New case	0.99	1.01	0.23	4.46
Recurrent	0.77	1.28	0.22	7.47
Treatment after failure	0.40	2.21	0.34	14.25
Treatment after loss to follow-up	0.51	0.49	0.06	4.02
Previously treated	Ref	-	-	-
Comorbidity				
None	0.32	1.39	0.73	2.65
Present	Ref	-	-	-
Site-wise classification of TB				
EPTB	0.71	1.12	0.55	2.40
PTB	Ref	-	-	-

Predictors of Treatment Success (vs. Death)

After adjusting for potential confounders, geriatric age was a significant and strong independent predictor of reduced likelihood of treatment success, compared to death. Geriatric patients had approximately 85% lower odds of achieving treatment success relative to non-geriatric patients (aOR = 0.148; 95% CI: 0.076-0.289; p < 0.001). HIV status was also a significant predictor. Patients with non-reactive HIV status had markedly higher odds of treatment success compared to those with unknown HIV status (aOR = 4.644; 95% CI: 2.459-8.772; p < 0.001), suggesting that known and negative HIV status is associated with better outcomes. Reactive HIV status also showed a numerically higher odds ratio (aOR = 4.602), though this did not reach statistical significance (p = 0.160), likely due to small cell counts. Other covariates, including sex, weight, socioeconomic status, diabetes status, programmatic classification of TB cases, comorbidities, and site of disease, did not show statistically significant associations with treatment success in the adjusted model.

Predictors of Treatment Failure/LFU (vs. Death)

In the comparison of treatment failure or LFU against death, geriatric age did not emerge as a statistically significant predictor (aOR = 0.54; 95% CI: 0.25-1.16; p = 0.12). This suggests that while geriatric patients are at substantially higher risk of death relative to treatment success, the distinction between dying and being LFU is not significantly driven by age group alone in the adjusted model. None of the other covariates, including sex, weight, socioeconomic status, HIV status, diabetes, programmatic case classification, comorbidities, or site of TB, reached statistical significance for this outcome comparison.

## Discussion

The impact of ageing on geriatric disorders has been extensively studied; however, its influence on infectious diseases, particularly TB, remains comparatively underexplored. Infection-related morbidity in older adults may result not only from pathogen burden but also from impaired host responses to inflammation-induced tissue injury and a diminished ability to resolve inflammatory damage [12]. TB continues to be the leading infectious cause of mortality worldwide, disproportionately affecting the elderly due to mechanisms such as immunosenescence and the chronic pro-inflammatory state termed “inflammaging,” although these were not directly measured in our study and are discussed as theoretical constructs supported by existing literature [13]. Age-related impairments in T-cell immunity and reduced hypersensitivity responses further increase susceptibility to TB. Successful therapy in this population critically depends on strict adherence to anti-tubercular treatment; however, elderly patients remain at higher risk of treatment failure and incomplete recovery [14]. In this context, the present study provides contemporary programmatic data to inform strategies for improving TB outcomes in the geriatric population. 

This study demonstrates substantial demographic, clinical, radiographic, and outcome disparities between geriatric and non-geriatric TB patients within the NTEP setting. The pronounced male predominance observed in the geriatric group (68.8%) is consistent with findings from previous Indian and international studies, including Mukherjee et al., and aligns with national TB data, which reported nearly threefold higher TB detection rates among males. Gender differences have also been described in disease presentation, clinical forms of TB, treatment adherence, and cure rates among patients receiving anti-tubercular therapy [15,16]. This male predominance in older adults likely reflects the cumulative effects of lifelong occupational exposure, a higher prevalence of smoking and other lifestyle-related risk factors, and gender-based disparities in healthcare access, awareness, and utilization. Collectively, these observations highlight the need for age- and gender-responsive TB control strategies that address underlying social determinants of health in the geriatric population.

The higher burden of comorbidities among elderly TB patients - particularly DM (35.3%), hypertension (18.5%), asthma (7.2%), and ischemic heart disease (5.5%) - is consistent with previous evidence highlighting the bidirectional relationship between chronic diseases and TB susceptibility. Jeon and Murray reported a significantly increased risk of TB among individuals with diabetes, while Dooley and Chaisson emphasized its influence on disease presentation and treatment response, with TB itself capable of inducing glucose intolerance and worsening glycemic control [17,18]. Comorbid conditions in older adults not only increase the risk of developing active TB but also complicate disease progression, reduce treatment efficacy, and predispose patients to adverse outcomes. For instance, DM has been shown to impair innate and adaptive immune responses, thereby increasing the likelihood of severe pulmonary involvement and delayed sputum conversion. Similarly, cardiovascular comorbidities can limit physiological reserves and contribute to poor tolerance of anti-TB pharmacotherapy, further amplifying the risk of treatment failure and mortality. These findings underscore the critical importance of integrated care approaches that address both TB and chronic comorbidities in geriatric populations.

The higher frequency of pulmonary TB among elderly patients in our cohort mirrors observations from other high-burden settings [19,20]. While age-related immunosenescence is known to reduce cell-mediated immunity and impair the containment of latent infection, thereby increasing susceptibility to pulmonary reactivation, these mechanisms were not directly assessed in our study and should be interpreted as plausible theoretical explanations supported by existing literature. Similarly, chronic pathological lung changes common in older adults, such as fibrosis, emphysema, and bronchiectasis, may further predispose to pulmonary disease, although these factors were not systematically evaluated. Conversely, the relatively higher occurrence of extrapulmonary TB in younger patients may reflect stronger immune responses, greater diagnostic vigilance, and earlier healthcare-seeking behavior; however, these interpretations remain hypothetical [21]. These age-related differences in disease localization should, therefore, be viewed as associative findings, and the underlying mechanisms warrant further investigation in prospective studies.

Radiographic differences between geriatric and non-geriatric TB patients were prominent in our cohort. Elderly patients more commonly exhibited extensive parenchymal lung involvement, including bronchiectasis, diffuse infiltrates, and cavitary lesions, suggesting delayed healthcare seeking, a higher burden of reactivation TB, and the presence of underlying chronic lung disease [22]. These patterns are typical of long-standing or advanced pulmonary pathology and are often associated with more severe disease and poorer outcomes in older adults. In contrast, non-geriatric patients more frequently demonstrated radiological features such as lymphadenopathy, focal consolidation, and healed fibrotic nodules, which are characteristic of primary TB infection or recent exposure. Woodring et al. [23] highlighted several radiographic pitfalls contributing to missed TB diagnoses in adults, including failure to recognize hilar or mediastinal lymphadenopathy, misinterpretation of minimal fibroproductive lesions as inactive disease, and overlooking healed sequelae or atypical lesion distributions. Such diagnostic challenges are particularly relevant in elderly patients, in whom radiographic findings may be subtle, atypical, or confounded by pre-existing lung pathology. These observations underscore the importance of integrating radiographic evaluation with clinical and microbiological findings to support age-specific diagnostic and management strategies, with older adults often requiring closer monitoring and more intensive supportive care to reduce complications.

Treatment outcomes among geriatric TB patients in our cohort were markedly poorer than in younger individuals, with nearly threefold higher mortality (17.3% vs. 3.3%) and substantially lower treatment success rates (62.0% vs. 87.8%), consistent with findings from other cohort studies [24,25]. These adverse outcomes may reflect a convergence of biological, clinical, and social factors; however, variables such as frailty, multimorbidity burden, polypharmacy, nutritional status, and delays in diagnosis were not directly measured in our study, and should be interpreted as plausible explanatory mechanisms supported by existing literature rather than confirmed findings [26]. Interestingly, treatment interruptions were not observed among elderly patients; while this may suggest better adherence or caregiver support, it could also be influenced by higher on-treatment mortality. Although the WHO’s TB control strategy has improved cure rates, its broader epidemiological impact remains limited, highlighting the need for further research into population-level determinants and targeted interventions for vulnerable groups, such as the elderly.

The present multinomial logistic regression underscores the profound impact of ageing on TB outcomes. In our cohort, geriatric patients had significantly lower odds of treatment success compared to death (aOR = 0.148; 95% CI: 0.076-0.289; p < 0.001), indicating nearly an 85% reduction in the likelihood of favorable outcomes. This finding is consistent with global evidence from the WHO, which reports increased mortality among elderly TB patients due to factors such as immunosenescence, delayed diagnosis, and higher comorbidity burden [1]. Similar observations were reported by Negin et al., demonstrating poorer treatment outcomes in older populations [15]. Furthermore, Tabernero et al. [27] found that advanced age was strongly associated with mortality, but not with LFU, aligning with our finding that geriatric age was not a significant predictor of treatment failure/LFU (aOR = 0.54; 95% CI: 0.25-1.16; p = 0.12). Additionally, the significantly higher odds of treatment success among patients with non-reactive HIV status (aOR = 4.644; p < 0.001) emphasize the prognostic importance of known HIV status, possibly reflecting better healthcare engagement and timely management. These findings are further supported by prior studies, including Tabernero et al. [27], and large cohort analyses from Uzbekistan [28] and India [9], all of which consistently demonstrate poorer outcomes and higher mortality among elderly TB patients, reinforcing the need for focused attention on this vulnerable population.

**Figure 4:**
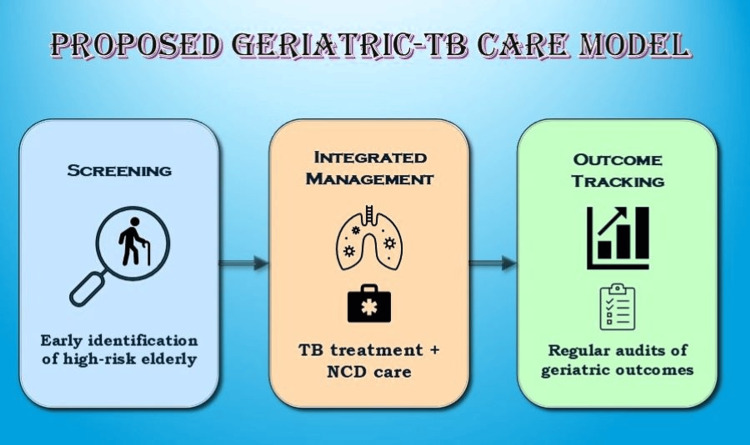
Proposed Geriatric TB Care Model Schematic representation of an integrated, patient-centric geriatric TB care model, emphasizing early screening, comprehensive geriatric assessment, comorbidity management, treatment adherence monitoring, and multidisciplinary follow-up to improve treatment outcomes and quality of life among elderly TB patients. This image is an original, author-created schematic developed using Microsoft PowerPoint 2024 (Microsoft Corporation, Redmond, WA, USA) and was not generated using AI. TB: Tuberculosis; NCD: Non-communicable Diseases

From a programmatic and policy standpoint, these findings highlight the need to strengthen geriatric-focused TB control strategies within the NTEP framework. Focused screening of high-risk elderly populations, early identification and optimal management of comorbidities, and closer integration of TB services with existing chronic disease care programs are essential to improving outcomes. Provision of age-appropriate adherence support and follow-up mechanisms may further reduce mortality and prevent LFU in this vulnerable group. In parallel, targeted awareness initiatives promoting early symptom recognition and timely healthcare-seeking behavior among older adults could enable earlier diagnosis and treatment initiation. A proposed model for comprehensive geriatric TB care is illustrated in Figure 4.

This study’s strengths include its robust comparative design, relatively large sample size, and comprehensive evaluation of demographic, clinical, radiographic, and treatment outcome variables, which together provide a detailed understanding of age-related differences in TB presentation and management. This study has several limitations that should be considered while interpreting the findings. Its single-center, retrospective design, using routinely collected NTEP and NIKSHAY data, may limit generalizability and introduce information bias due to incomplete or inconsistent documentation. Outcomes such as treatment success and mortality may be misclassified, and the observational design precludes causal inference. Important confounders - including nutritional status (BMI), anemia severity, frailty indices, socioeconomic determinants, drug-resistance patterns, and detailed sputum bacillary load - were not consistently available. Absence of height data prevented BMI calculation, and lack of time-to-death data precluded survival analysis. Radiological findings were based on routine clinician documentation rather than standardized reporting, potentially leading to misclassification. Small cell counts in certain variables limited inclusion in adjusted models, and interaction analyses were not feasible due to instability. Group imbalance and short study duration may have affected statistical power and limited the assessment of seasonal and long-term outcomes. Additionally, a high proportion of “unknown” values (e.g., HIV status) may introduce non-random missing data bias. Despite these limitations, the study provides valuable, real-world, programmatic insights into TB outcomes.

In conclusion, geriatric TB patients in this Indian NTEP cohort experience higher comorbidity burdens, more severe pulmonary disease, and poorer treatment outcomes compared to younger adults. These findings highlight the critical need for age-specific TB strategies, including integrated management of chronic diseases, tailored adherence support, and enhanced surveillance for early diagnosis. Incorporating these approaches into national and global TB programs will be essential to reduce mortality, improve treatment success, and achieve equitable TB control across all age groups.

## Conclusions

Geriatric TB patients in our cohort demonstrated distinct demographic, clinical, radiographic, and outcome patterns compared to younger adults, with a higher comorbidity burden and poorer treatment outcomes, including increased mortality. In contrast, non-geriatric patients more frequently presented with extrapulmonary TB and achieved higher treatment success. These findings, derived from both descriptive analyses and adjusted multinomial regression, highlight significant associations between geriatric age, clinical characteristics, and treatment outcomes within the NTEP framework.

However, given the observational design, these results should be interpreted as associations rather than causal relationships, and factors such as diagnostic delay and disease severity require further investigation. While our study robustly demonstrates increased clinical complexity in elderly TB patients, proposed strategies - including integration of TB and chronic disease care, enhanced screening, and a "Geriatric-focused TB care model" - should be considered hypothesis-generating, evidence-informed recommendations that warrant validation through prospective and implementation research.
